# Prevalence and risk factors of depression in patients with diabetes mellitus: a systematic review and meta-analysis

**DOI:** 10.3389/fendo.2025.1660478

**Published:** 2025-10-20

**Authors:** Ke Yang, Yuyang Fang, Junbo He, Jing Li

**Affiliations:** ^1^ Dongzhimen Hospital, Beijing University of Chinese Medicine, Beijing, China; ^2^ Beijing University of Chinese Medicine, Beijing, China

**Keywords:** diabetes mellitus, depression, prevalence, risk factors, mental health

## Abstract

**Objective:**

This systematic review and meta-analysis examined the prevalence of depression among individuals with diabetes and identified associated risk factors.

**Methods:**

Five databases (PubMed, Web of Science, Cochrane, ProQuest, Embase) were searched for observational studies reporting depression prevalence and multivariable-adjusted risk factors in diabetic populations. Two reviewers independently screened and extracted data. Analyses were conducted using R software.

**Results:**

Thirty-nine studies involving 17,486 diabetic patients were included. The pooled prevalence of depression was 35% (95% CI: 30%–41%). Risk factors included age ≤60 years, female sex, being single, unemployment, physical inactivity, anxiety, limited social support, poor medication adherence, complications (neuropathy, nephropathy, retinopathy, foot ulcers), physical disability, insulin therapy, combined insulin–oral treatment, and fasting glucose ≥126 mg/dL.

**Conclusion:**

Depression affects over one-third of diabetic patients and is associated with sociodemographic, psychological, and clinical factors. Our study provides updated global evidence and identifies specific high-risk profiles (e.g., females, those with complications, or on combination therapy), supporting the need for targeted screening beyond general recommendations. These findings support the integration of standardized depression screening tools such as the PHQ-9 into routine diabetes care, particularly in resource-limited settings. For patients with identified risk factors, regular follow-up screening is recommended to enable early detection and timely intervention. Routine screening and timely intervention are essential, especially for high-risk groups. Longitudinal studies are needed to clarify causal links and inform targeted prevention.

**Systematic review registration:**

https://www.crd.york.ac.uk/prospero/, identifier CRD420250656589.

## 1 Introduction

Diabetes mellitus is a global health challenge, with prevalence projected to rise to 783 million adults by 2045 (1). Beyond its physical health and economic impacts (2, 3), diabetes imposes a significant psychological burden, as evidenced by its frequent co-occurrence with depression. This dual burden exacerbates disease management and worsens clinical outcomes.

Depression, defined as at least two weeks of low mood or reduced interest that impairs functioning (4), is frequently comorbid with diabetes. The two conditions share biological mechanisms, including CPE gene dysfunction (5), inflammatory pathways (6), and HPA axis dysregulation (7). Interventions such as anti-inflammatory diets and acupuncture have shown benefits for both HbA1c and depressive symptoms (8). Compared to diabetes alone, comorbidity is linked to greater glucose variability (9), poor adherence (10), and higher vascular risk (11). A 2024 UK cohort study reported that major depressive disorder accounted for 7.8% of new vascular events, and depressive symptoms for 3.8% (12).

Although numerous meta-analyses exist, most focus on single regions, reporting varied prevalence—e.g., 42% in Bangladesh (13), 34.6% in Ethiopia (14), and 25.9% in China (15)—reflecting differences in healthcare access and sociodemographic factors. Many lack multivariable analyses and fail to adjust for confounders (16, 17), limiting comparability. This study updates the global prevalence and integrates data from multiple countries to construct a multilevel model of sociodemographic, psychosocial, clinical, and biochemical correlates, offering evidence to support precise, targeted interventions.

## 2 Methods

This meta-analysis adhered to PRISMA guidelines (Appendix 1) and was prospectively registered in PROSPERO (CRD420250656589). A systematic search of PubMed, Web of Science, Cochrane Library, ProQuest, and Embase was performed to locate studies on depression prevalence and related risk factors among adults with diabetes. Both subject terms and free-text terms were employed. The initial search was performed between February 28 and March 7, 2025, and updated on June 17, 2025. Full search strategies are presented in Appendix 2.

Studies were eligible if they met the following criteria (1): observational design (cross-sectional or cohort) (2); published in English (3); participants aged 18 years or older (4); reported depression prevalence and risk estimates (ORs with 95% CIs, or data sufficient for calculation); and (5) utilized validated depression assessment tools. Exclusion criteria included: lack of full text, duplicate records, incomplete data, non-English language, or poor methodological quality.

All records were imported into EndNote 21 to remove duplicates. Titles and abstracts were independently screened by two reviewers, followed by evaluation of full texts. Data extracted comprised study title, author, publication year, design, setting, sample size, number of depression cases, prevalence and related influencing factors. Any disagreements were settled through discussion or consultation with a third reviewer.

Methodological quality was appraised using JBI checklists for prevalence and analytical cross-sectional studies. Items were scored as “yes,” “no,” or “unclear,” with “yes” responses assigned 1 point. Based on the proportion of positive responses, studies were categorized as high (≤49%), moderate (50–69%), or low (≥70%) risk. Studies deemed high risk on both tools were excluded.

Data were organized in Microsoft Excel and analyzed using R software (version 4.4.3) with the meta, metafor, dplyr, and metaprop packages. The Freeman–Tukey double arcsine transformation (sm = “PFT”) was used to stabilize variances in prevalence estimates. Between-study heterogeneity was assessed using the DerSimonian–Laird method (method.tau = “DL”). Subgroup analyses and pooled adjusted odds ratios (AORs) were calculated using random-effects models with restricted maximum likelihood estimation (REML), which improves precision in small samples or when heterogeneity is pronounced. The Hartung–Knapp adjustment was applied for random-effects confidence intervals, except in cases involving only two studies or low heterogeneity (I² < 50%), where standard methods were retained to prevent overly conservative intervals.All R code used for the meta-analyses is available for review at: https://dedi-meta.github.io/, ensuring full transparency and reproducibility.

Heterogeneity was assessed using Cochran’s Q test and the I² statistic, with P < 0.1 and I² > 50% considered indicative of substantial heterogeneity. To explore the sources of this heterogeneity, we pre-specified two strategies: subgroup analyses and multivariate meta-regression.

Subgroup analyses were stratified by age, sex, geographic region, publication year, study duration, setting, and depression measurement tools. Additionally, multivariate meta-regression was pre-planned to examine the potential moderating effects of study-level mean age, survey year, and geographic region on the prevalence estimates, as these variables represent key sources of clinical and methodological heterogeneity. Risk factors were synthesized using AORs derived from multivariable logistic regression. Heterogeneity was assessed using Cochran’s Q test and the I² statistic, with P < 0.1 and I² > 50% considered indicative of substantial heterogeneity. Inter-rater agreement for quality assessment was measured by Cohen’s kappa coefficient. Publication bias was assessed using Begg’s and Egger’s tests.To assess the robustness of the results, a leave-one-out sensitivity analysis was carried out. The results of these analyses were visually presented using forest plots.

## 3 Results

**Figure 1:**
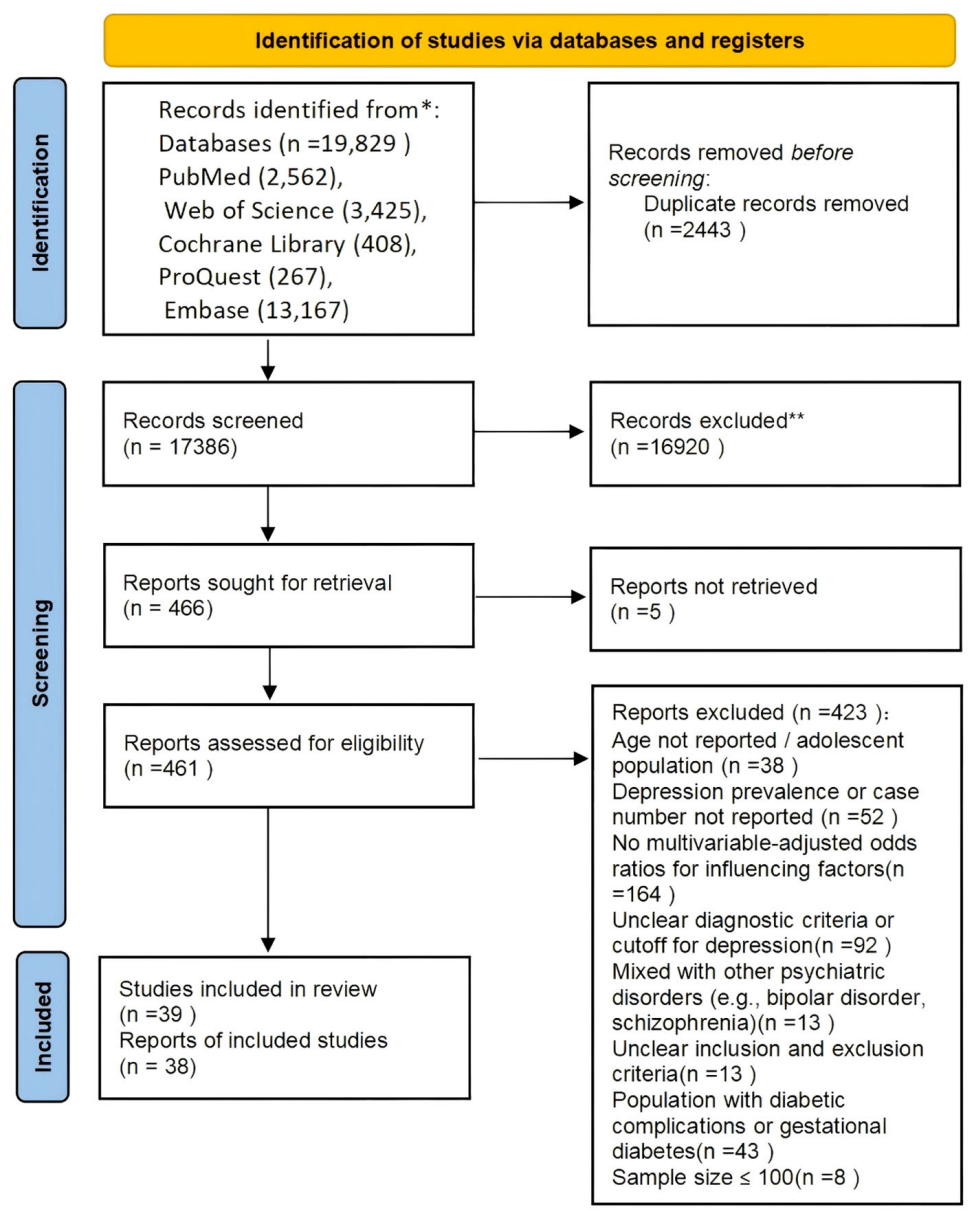
PRISMA 2020 flowchart depicting the study selection process.

A total of 19,829 articles were retrieved from five databases: PubMed (2,562), Web of Science (3,425), Cochrane (408), ProQuest (267), and Embase (13,167). After removing duplicates in EndNote 21, 17,386 unique records remained. Following title and abstract screening, 466 articles were selected for full-text review. Full texts of 5 articles could not be retrieved, leaving 461 articles for full-text screening. Of these, 423 were excluded for reasons such as unclear inclusion and exclusion criteria, unclear diagnostic criteria, lack of multivariate analysis, or inclusion of ineligible populations. Ultimately, 38 articles met the inclusion criteria. One study separately analyzed two independent regional datasets, resulting in a total of 39 included studies (**Figure 1**).

These studies were all cross-sectional in design and involved 17,486 diabetic patients. They were published between 2007 and 2025, with study durations ranging from 1 to 12 months. Geographically, the studies were conducted in Asia (22 items), Africa (11 items), Europe (2 items), and North America (4 items). Sample sizes ranged from 148 to 2,182 participants. All studies underwent dual quality assessment using the JBI tools: JBI 1 for prevalence studies and JBI 2 for analytical cross-sectional studies. All were rated low risk in the JBI 2 assessment; five were rated high risk in JBI 1(**Table 1**). Inter-rater agreement was assessed to ensure consistency in quality ratings. For JBI 1, the unweighted Cohen’s kappa was 0.891, reflecting high concordance. No discrepancies were found with JBI 2, so further statistical analysis was unnecessary.

**Table 1:** Summary of characteristics of included studies. (Full details of the 39 included studies are provided in Appendix 3).

Author + year	Country	N	Assessment	Prevalence (%)
Mohamed Abd-Elgawad2023	Egypt	679	HADS≥8	34.17
Shahad Abduljalil Abualhamael2024	KSA	251	DASS-21≥10	49.40
Hesham Abuhegazy2022	KSA	350	PHQ-9>10	36.57
Seid Yimam Ali	Ethiopia	263	PHQ-9≥5	47.15
Abdullahi S. Aminu2017	India	200	PHQ-9≥5	37.50
Muhammad Atif2018	Pakistan	400	GDS-15≥5	67.50
Gedion Asnake Azeze2020	Ethiopia	410	PHQ-9≥5	29.27
anteneh Messele Birhanu2016	Ethiopia	415	PHQ-9≥5	15.42
Habtamu Birhanu2022	Ethiopia	310	PHQ-9≥10	41.61
Tania Dehesh2020	Iran	1500	BDI-II≥18	59.00
Mohamed Ebrahim2021	Ethiopia	401	PHQ-9≥5	48.88
Mohamed Hassan Elnaem2025	Indonesia/Malaysia	606	PHQ-9≥10	56.60
Nigus Alemnew Engidaw2020	Ethiopia	403	PHQ-9≥5	21.34
Annie C. H. Fung2018	China	325	GDS-15≥7	12.92
Malgorzata Gorska-Ciebiada2014	Poland	276	GDS-30≥10	29.71
Sheikh Mohammed Shariful Islam2015	Bangladesh	515	PHQ-9≥5	61.94
Firdous Jahan2011	Pakistan	320	self-reported validated questionnaire≥9	17.50
Mihyun Jeong2021	Korean	1529	PHQ-9≥10	9.74
Ashmita Karki2024	Nepal	481	PHQ-9≥5	25.57
Kankana Karpha2022	India	152	PHQ-9≥5	39.47
Nuket Bayram Kayar2017	Turkey	154	SCID-I scale	18.18
Steven M. Kogan2007	America	200	CES-D≥16	36.00
Rehanguli Maimaitituerxun2023	China	496	HADS-D≥8	27.22
Makda Abate Belew2023	Ethiopia	426	PHQ-9≥5	47.65
Eva O. Melin2017	Sweden	148	HADS-D≥8	11.49
Nelda Mier2008①	Mexico	200	CES-D≥16	40.50
Nelda Mier2008②	America	172	CES-D≥16	38.95
Nur Adam Mohamed2024	Somalia	360	DASS-21≥10	44.72
Lili Husniati Yaacob2012	Malaysia	260	HADS-D≥9	20.77
Mussa R. Mussa2023	Tanzania	267	PHQ-9≥5	72.66
Kabtamu Nigussie2023	Ethiopia	416	HADS≥8	42.31
Hina Sharif2023	Pakistan	493	PHQ-9≥5	30.83
Avinash K. Sunny2019	Nepal	278	BDI-II≥16	22.66
Waleed M Sweileh2014	Palestine	294	BDI-II≥16	40.82
Thitiphan Thaneerat2009	Thailand	250	HADS-D≥8	28.00
Nhu Minh Hang Tran2021	Vietnam	216	PHQ-9≥10	23.15
Allan Oliver Dampil2019	Philippines	476	PHQ-9≥5	81.09
Yiting Wang2016	America	2182	PHQ-9≥10	11.73
Weijun Zhang2015	China	412	BDI-II≥14	34.47

N, Sample Size; Assessment, Depression Assessment and Cut-off Score.

### 3.1 Prevalence of depression in diabetic patients

**Figure 2:**
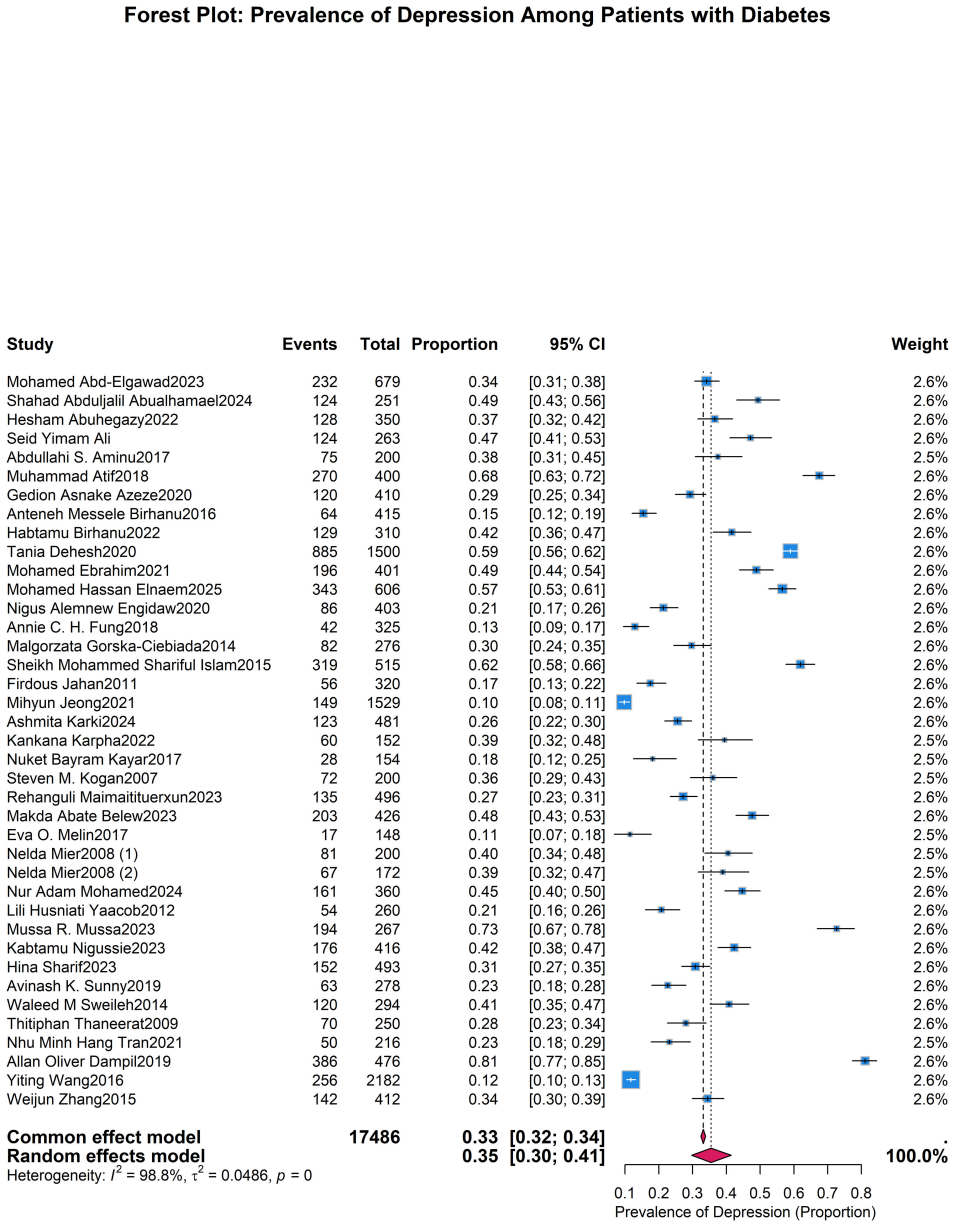
Forest plot presenting the pooled prevalence of depression in individuals with diabetes mellitus.

The pooled prevalence of depression among individuals with diabetes was 35% (95% CI: 30%–41%), based on a random-effects model. Substantial heterogeneity was observed across studies (I² = 98.8%, τ² = 0.0351, P < 0.0001) (**Figure 2**). Publication bias was evaluated using Begg’s and Egger’s tests, with no significant bias detected (Begg’s p = 0.5778; Egger’s p = 0.1351). The non-significant results of Begg’s and Egger’s tests suggest no substantial publication bias was detected.

### 3.2 Subgroup analyses

**Figure 3:**
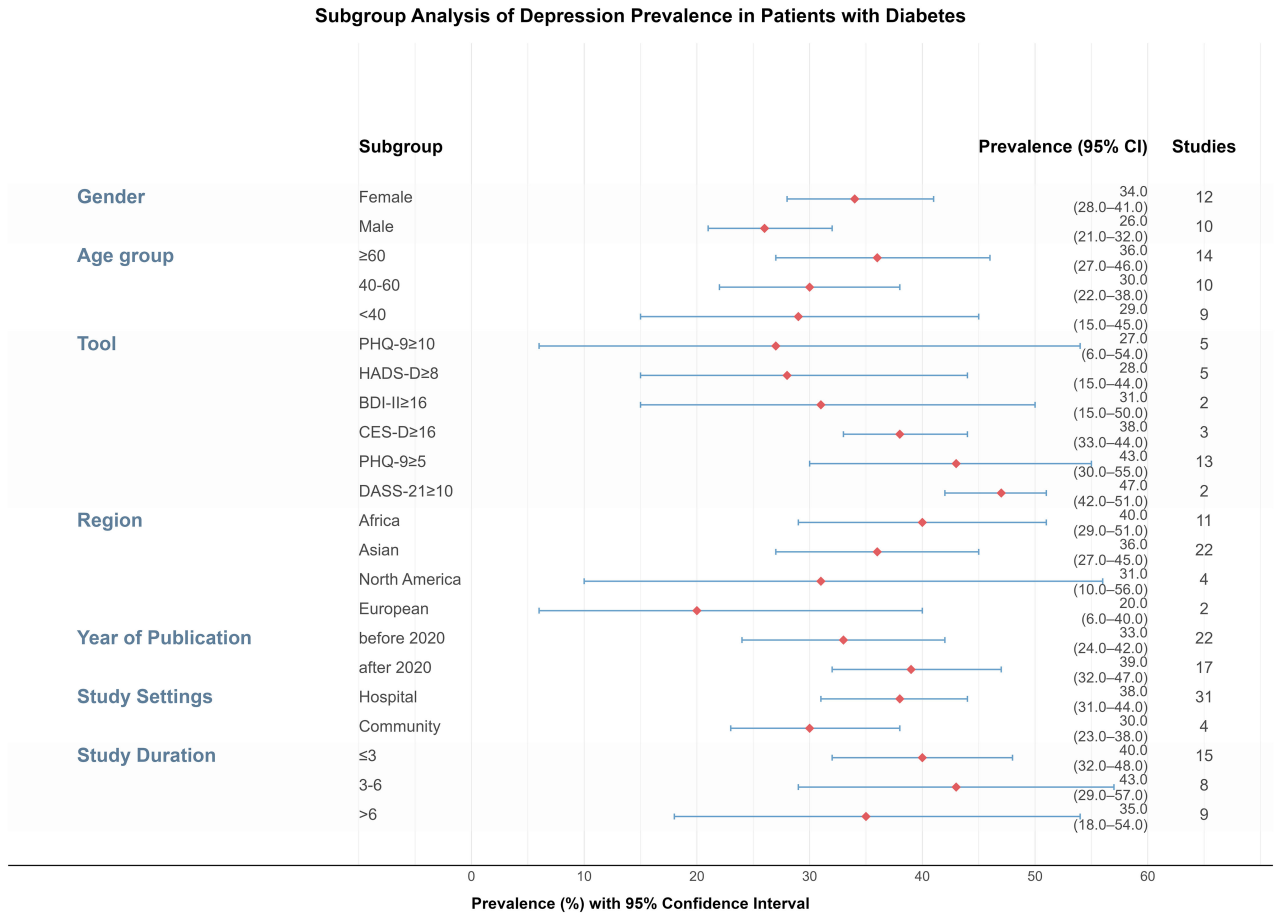
Forest plot of subgroup analysis on depression prevalence in individuals with diabetes.

To explore sources of heterogeneity, subgroup analyses were conducted based on age, gender, publication year, study duration, setting, geographic region, and depression assessment tools (**Figure 3**).

Age: Depression prevalence increased slightly with age: 29% (under 40) (19–27), 30% (40–60) (19–28), and 36% (≥60) (19–29, 38, 46, 47). However, differences among age groups were not statistically significant (P = 0.4906). Heterogeneity remained high within subgroups (I² ≥ 85.9%).

Gender (18, 19, 21–28, 30, 32, 33, 36, 38, 39, 46–55): The prevalence was higher among female patients (34%, 95% CI: 28%–41%) than male patients (26%, 95% CI: 21%–32%), with a statistically significant difference (P = 0.0392). The pooled OR for females compared to males was 1.51 (95% CI: 1.31–1.74), with moderate between-study heterogeneity (I² = 59%).

Depression Screening Tools/Cut-off Values: Six subgroup categories were formed based on tools and cut-offs used in at least two studies. The highest prevalence was found with DASS-21 ≥10 (32, 56) (47%, 95% CI: 19%–75%), and the lowest with PHQ-9 ≥10 (23, 38, 40, 47, 51) (27%, 95% CI: 6%–54%). Differences across tools were statistically significant (P = 0.0041).

Study Settings: Among the 36 studies with available setting information, 31 (19–23, 25, 27–34, 36–39, 46, 48–50, 52–59) were hospital-based and 5 (18, 24, 26, 35, 60) community-based. Depression prevalence was higher in hospital settings (38%, 95% CI: 31%–44%) compared to community settings (30%, 95% CI: 23%–38%), though this difference was not statistically significant (P = 0.0856).

Study Duration: Of the 32 studies with duration data, 15 (20–23, 25, 27, 29, 32, 35, 48–50, 57–59) had a duration ≤3 months, 8 (18, 19, 33, 34, 36, 37, 51, 56) lasted 3–6 months, and 9 (26, 28, 30, 31, 38, 39, 46, 52) were >6 months. Prevalence was highest in studies with durations of 3–6 months (43%) and lowest in studies longer than 6 months (35%). However, no significant difference was observed among groups (P = 0.7340).

Region: Studies conducted in Africa (20–23, 25, 29, 32, 34, 48, 50, 58) showed the highest pooled prevalence (40%, 95% CI: 29%–51%), followed by Asia (20, 24, 46, 47, 51, 53, 54, 57, 59, 60) (36%), North America (31, 40, 60) (31%), and Europe (30, 53) (20%). Although point estimates varied, the differences were not statistically significant (P = 0.3092), and heterogeneity within regions remained high.

Year of Publication: Depression prevalence was slightly higher in studies published after 2020 (19, 20, 23–25, 28, 29, 32, 34, 35, 38, 47, 48, 50, 51, 56, 59) (39%) compared to those before 2020 (18, 21–23, 26, 27, 30, 31, 33, 36, 37, 39, 40, 46, 49, 52–55, 57, 58, 60) (33%), but the difference was not statistically significant (P = 0.2354).

This study performed a multivariate meta-regression on mean age, survey year, and region. Results showed no significant effects of age (β = -0.0006, p = 0.921), survey year (β = 0.0118, p = 0.178), or region (QM = 3.83, p = 0.574) on depression prevalence in diabetic patients (Appendix 4).

### 3.3 Sensitivity analysis

A leave-one-out sensitivity analysis was conducted to evaluate the impact of each study on the overall prevalence estimate. Exclusion of individual studies did not significantly alter the pooled prevalence, which remained stable between 35% (95% CI: 30%–40%) and 37% (95% CI: 32%–42%). The overall effect estimate (theta = 0.35) remained within the 95% CIs of all iterations. The stability of the pooled prevalence upon successive exclusion of each study indicates that the overall result was not disproportionately influenced by any individual study (Appendix 5).

### 3.4 Influencing factors

**Figure 4:**
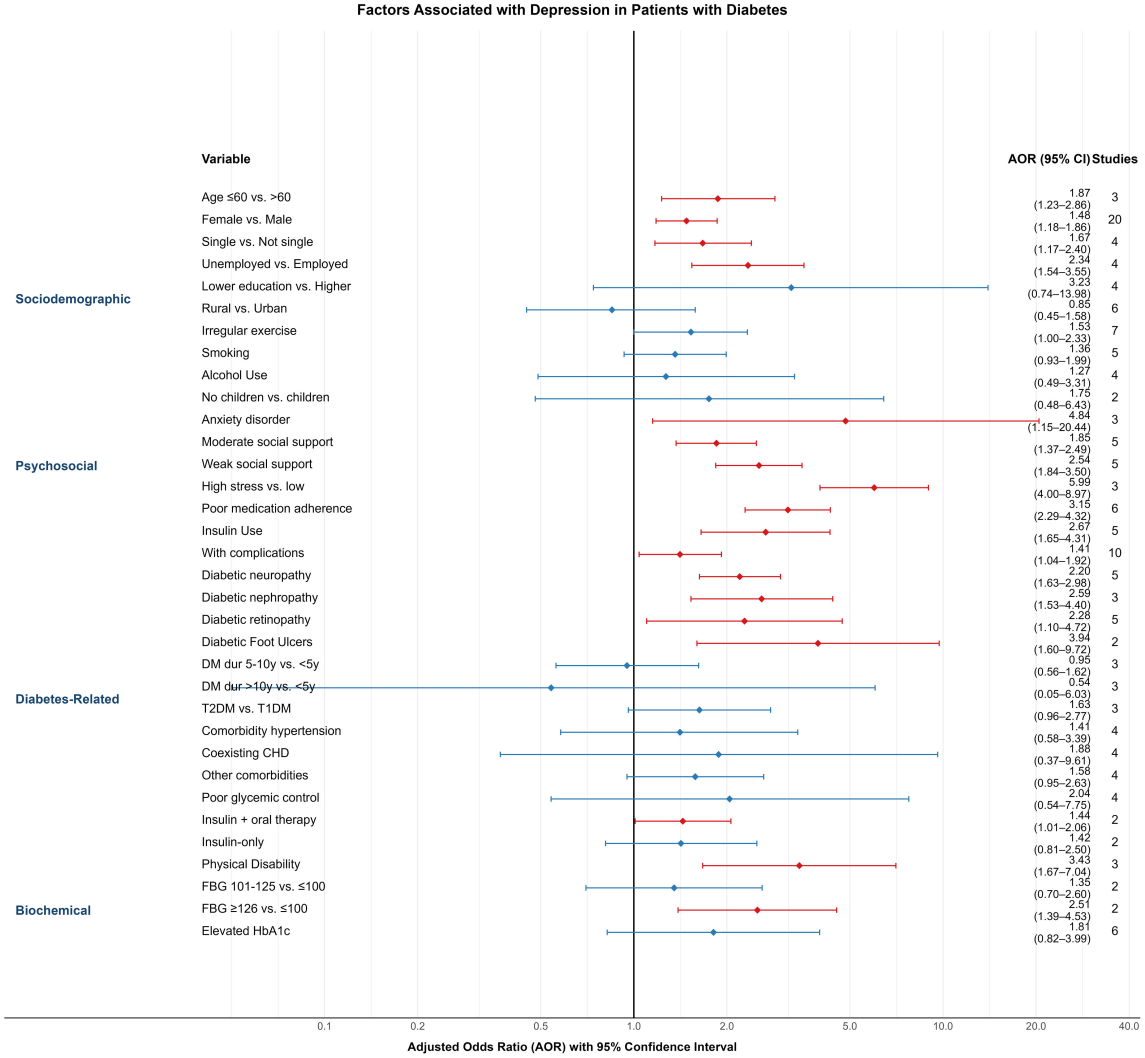
Forest plot of variables linked to depression prevalence among individuals with diabetes.

In this meta-analysis, 30 variables were identified as potential factors influencing depression among diabetic patients. These were categorized into four domains: sociodemographic characteristics, psychosocial conditions, diabetes-related factors, and biochemical indicators. All included variables were derived from multivariable logistic regression models, ensuring adjustment for potential confounders. The following summarizes the pooled results (**Figure 4**).

#### 3.4.1 Sociodemographic factors

1. Age: Three studies (28, 38, 46) reported age as a predictor of depression. Patients aged ≤60 years had a significantly higher risk compared to those >60 years (AOR = 1.87; 95% CI: 1.23–2.86).
2. Gender: Based on 20 studies (18, 22, 23, 25–27, 31, 32, 34, 36, 39, 40, 46, 47, 49–51, 53, 56, 60), female diabetic patients had a higher likelihood of depression than males (AOR = 1.48; 95% CI: 1.18–1.86).
3. Marital Status: In four studies (22, 27, 53, 57), being single was associated with increased depression risk compared to being married or in a partnership (AOR = 1.67; 95% CI: 1.17–2.40).
4. Employment Status: Four studies (18, 27, 36, 47) found that unemployed individuals had a higher risk of depression than those employed (AOR = 2.34; 95% CI: 1.54–3.55).
5. Educational Attainment (26, 27, 31, 37): Lower education levels were associated with a non-significantly elevated risk (AOR = 3.23; 95% CI: 0.74–13.98).
6. Place of Residence: Six studies (18, 22, 24, 35, 51, 58) examined urban–rural differences. Patients living in rural areas had a slightly lower—but non-significant—risk of depression compared to urban counterparts (AOR = 0.85; 95% CI: 0.45–1.58).
7. Exercise Habits: Seven studies (22, 23, 32, 49, 51, 53, 56) reported that irregular physical activity was linked to increased depression risk (AOR = 1.53; 95% CI: 1.00–2.33).
8. Smoking and Alcohol Use: While both behaviors showed trends toward higher depression risk—smoking (24, 27, 49, 53, 56) (AOR = 1.36; 95% CI: 0.93–1.99) and alcohol use (23, 27, 29, 49) (AOR = 1.27; 95% CI: 0.49–3.31)—the results were not statistically significant.
9. Children: In two studies (21, 22), patients without children had a higher, but non-significant, risk of depression (AOR = 1.75; 95% CI: 0.48–6.43).

#### 3.4.2 Psychosocial factors

1. Anxiety Disorder: Three studies (30, 37, 48) found a strong association between comorbid anxiety and depression in diabetic patients (AOR = 4.84; 95% CI: 1.15–20.44).
2. Social Support: Five studies (23, 25, 35, 50, 58) assessed social support levels. Compared to high support, moderate (AOR = 1.85; 95% CI: 1.37–2.49) and low support (AOR = 2.54; 95% CI: 1.84–3.50) were significantly associated with higher depression risk.
3. Stress (34, 38, 47): High perceived stress was significantly associated with depression (AOR = 5.99; 95% CI: 4.00–8.97; 3 studies).
4. Medication Adherence (19, 34, 37, 38, 51, 56): Non-adherence was significantly associated with depression (AOR = 3.15; 95% CI: 2.29–4.32; 6 studies).

#### 3.4.3 Diabetes-related factors

(1) Insulin Use (26, 27, 53, 59, 60): Insulin-treated patients had significantly higher depression risk compared to those not using insulin (AOR = 2.67; 95% CI: 1.65–4.31).

(2) Complications (18, 20, 22, 27, 29, 33, 48–50, 57): The presence of diabetic complications was associated with higher depression risk (AOR = 1.41; 95% CI: 1.04–1.92; 10 studies), as were specific complications:

Neuropathy (19, 39, 48, 54, 59): AOR = 2.20 (95% CI: 1.63–2.98)
Nephropathy (37, 54, 59): AOR = 2.59 (95% CI: 1.53–4.40)
Retinopathy (21, 34, 39, 48, 59): AOR = 2.28 (95% CI: 1.10–4.72)
Foot Ulcers (22, 30): AOR = 3.94 (95% CI: 1.60–9.72)

(3) Duration of Diabetes (22, 29, 51): No statistically significant association was observed for disease duration (5–10 years: AOR = 0.95; >10 years: AOR = 0.54).

(4) Type of Diabetes (22, 30, 58): Type 2 diabetes was associated with a higher—but non-significant—risk compared to Type 1 (AOR = 1.63; 95% CI: 0.96–2.77).

(5) Comorbid Conditions:

Hypertension (21, 39, 47–49, 52, 54, 59) (AOR = 1.41; 95% CI: 0.58–3.39),
Coronary heart disease (30, 39, 47, 59) (AOR = 1.88; 95% CI: 0.37–9.61),
Other comorbidities (18, 22, 50, 52) (AOR = 1.58; 95% CI: 0.95–2.63),
were not significantly associated with depression.

(6) Glycemic Control (20–22, 39): Poor glycemic control was linked to higher, but non-significant, depression risk (AOR = 2.04; 95% CI: 0.54–7.75).

(7) Treatment Regimen (19, 49): Patients using both insulin and oral agents had a significantly higher depression risk (AOR = 1.44; 95% CI: 1.01–2.06), while insulin-only users did not (AOR = 1.42; 95% CI: 0.81–2.5).

(8) Physical Disability: Three studies (34, 35, 58) reported a strong association between disability and depression (AOR = 3.43; 95% CI: 1.67–7.04).

#### 3.4.4 Biochemical indicators

(1) Fasting Blood Glucose (35, 58): Patients with fasting glucose ≥126 mg/dL had significantly higher depression risk (AOR = 2.51; 95% CI: 1.39–4.53) compared to those with levels ≤100 mg/dL.

(2) HbA1c (27, 30, 49, 52, 56, 57): While elevated HbA1c levels were associated with a higher risk (AOR = 1.81; 95% CI: 0.82–3.99), the association was not statistically significant.

## 4 Discussion

Previous meta-analyses have firmly established the high comorbidity between depression and diabetes (41, 42). Our study builds upon this foundation by providing several critical advancements that refine our understanding and inform clinical practice. First, by including 39 cross-sectional studies from Asia, Africa, Europe, and North America (N = 17,486), we offer a more updated and geographically diverse synthesis. Our pooled prevalence estimate of 35% (95% CI: 30%–41%) is substantially higher than previous reports (41, 42), reflecting the contemporary and growing burden of this comorbidity. Second, and most importantly, unlike prior reviews that often relied on univariate analyses susceptible to confounding, our meta-analysis exclusively synthesizes evidence from multivariable-adjusted models. This methodological rigor allows us to identify a hierarchy of independent risk factors—spanning sociodemographic, psychosocial, and clinical domains—that persist after accounting for confounders. Consequently, our primary novel contribution lies in moving beyond the established recommendation for screening by providing the evidence necessary to implement stratified, risk-based screening protocols in clinical practice.

The high pooled prevalence underscores the substantial clinical burden. With the continuing rise in diabetes prevalence worldwide, the absolute number of individuals affected by both conditions will also grow, highlighting the urgent need for integrated care models that address their well-established bidirectional relationship (9, 11). The generalizability of our pooled estimate, however, should be considered in the context of the geographical distribution of the included studies, a point we expand upon in the Limitations section.

The pooled prevalence of depression in our diabetic cohort (35%) is substantially higher than estimates reported for the general global population, which typically range from 4% to 5% (43). This disparity underscores the immense psychological burden associated with diabetes. The etiological pathways are likely multifactorial, encompassing the relentless psychological stress of managing a chronic illness, the financial toxicity of treatment, disease-related stigma, and shared biological pathways such as chronic inflammation and HPA axis dysregulation (7, 8).

Furthermore, our analysis allows for a distinction between risk factors that are generalizable from the general population and those that may be amplified or more specific to the diabetic context. For instance, female gender is a well-established risk factor for depression in both general and diabetic populations, a finding corroborated in our study. In contrast, factors such as elevated fasting blood glucose, the presence of diabetes-specific complications (e.g., neuropathy), and insulin therapy appear to represent disease-specific amplifiers of depression risk. These factors likely contribute to the elevated prevalence observed in diabetes by interacting with underlying general vulnerabilities, creating a unique risk profile that necessitates tailored screening and intervention strategies.

A notable finding from our subgroup analysis was the higher pooled prevalence of depression in studies published after 2020 compared with earlier studies (39% vs 33%), although this difference did not reach statistical significance. While the cross-sectional design of the included studies limits causal inference, this temporal trend warrants attention. The onset of the COVID-19 pandemic in 2020 likely contributed to this increase. Patients with diabetes were particularly vulnerable to the pandemic’s multiple stressors, including elevated psychosocial burden (e.g., lockdowns, social isolation, financial insecurity), disruption of routine healthcare services, and a feedback loop whereby pandemic-related stress could worsen glycemic control, potentially amplifying depressive symptoms (44, 45). Additionally, heightened clinical and research focus on mental health during this period may have increased detection rates. If supported by future longitudinal studies, this pattern highlights the disproportionate mental health impact of global crises on vulnerable populations and emphasizes the need for healthcare systems to strengthen resilience and incorporate psychological support into chronic disease management.

Gender subgroup analysis showed a higher prevalence in females (34%) than males (26%), with an OR of 1.51 (95% CI: 1.31–1.74). This is consistent with broader evidence indicating that women have approximately double the risk of developing depression (61–64), potentially due to hormonal fluctuations (61), caregiving roles (62), emotional processing differences, and structural determinants such as the disproportionate burden of unpaid work (65). Routine screening in female patients is recommended.

Tool-based subgroup analysis showed highest prevalence with DASS-21 ≥ 10 (47%) and lowest with PHQ-9 ≥10 (27%). Differences may relate to timeframes (DASS-21: past week; PHQ-9: past two weeks), focus (subjective distress vs. functional impairment), and cultural responses—e.g., avoidance of suicide-related items in East Asian populations may lower PHQ-9 scores (66). These differences highlight the importance of culturally sensitive tool selection in clinical screening, and suggest that PHQ-9 may require adaptation or complementary methods in East Asian populations. Future research should adjust for such heterogeneity to improve comparability.

Multivariate meta-regression found no significant association between depression prevalence and mean age, survey year, or geographic region. Notably, this null finding is itself informative. It suggests that the drivers of heterogeneity are likely more complex and operate at a level not fully captured by these aggregate variables. Potential explanations include the preeminence of individual-level psychosocial and clinical factors (as identified in our risk factor analysis), nuanced cultural and socioeconomic differences that are obscured by broad regional categorizations, and fundamental methodological variations such as the use of different depression assessment tools. This aligns with other meta-analytic findings in psychiatric epidemiology (14, 67). Furthermore, this underscores the limitation of meta-regression (an ecological analysis) and highlights the necessity for future research utilizing individual patient data (IPD meta-analysis) to better elucidate these complex relationships.

Unlike most prior studies, our analysis included only factors adjusted by multivariable logistic regression. The following were associated with increased depression risk: age ≤60 years, female gender, single status, unemployment, physical inactivity, anxiety disorder, weak/moderate social support, poor medication adherence, complications (neuropathy, nephropathy, retinopathy, foot ulcers), physical disability, and fasting glucose ≥126 mg/dL. as well as treatment-related factors such as insulin use and combined oral and insulin therapy. Collectively, this set of independently associated factors provides a practical evidence base for the risk-stratified screening approach proposed in the introduction of this discussion.These findings suggest that clinicians should adopt individualized screening protocols, considering psychosocial and clinical risk profiles in routine practice.

Depression risk was higher in patients ≤60 years, consistent with prior finding (68). Younger patients may face greater life pressure and role burdens (69). Gender-related vulnerability was again confirmed, consistent with prior studies (15, 70). Single status also increased risk, likely due to reduced emotional and social support (15).

Unemployment was associated with elevated depression risk. A Taiwanese cohort study showed that employment reduced depressive symptoms by 32% over 3–4 years (71), consistent with other research (72). Depression and unemployment may interact bidirectionally through financial strain, loss of routine, and impaired work function.

Physical inactivity was another significant risk factor. Meta-analysis of 17 RCTs showed that physical activity significantly reduced depressive symptoms in T2DM patients (SMD = -0.57) (73). A 2025 cross-sectional study found that walking 4–7 days per week reduced poor mood likelihood by 57% (74). Mechanisms include increased BDNF, serotonin, and dopamine, and reduced inflammation (75). However, the observed association must be interpreted with caution due to the potential for reverse causality. While physical activity has consistently been shown to reduce the risk of depression (76), depressive symptoms such as anhedonia, fatigue, and diminished motivation may themselves lead to reduced engagement in physical activity. Longitudinal evidence supports this pathway; for example, Chen et al. (77) reported that depressive symptoms significantly predicted subsequent decreases in physical activity among older adults. These findings underscore the bidirectional nature of the relationship, suggesting a vicious cycle in which depression and physical inactivity reinforce one another. From a clinical perspective, this highlights the need for integrated management approaches that simultaneously address both mood disturbances and barriers to physical activity in patients with diabetes.

Psychosocial factors also played key roles. Anxiety, low social support, and poor medication adherence significantly increased depression risk. Anxiety may mediate the link between social support and depression, weakening the protective effect of support (78). Social support improves adherence and psychological resilience (79), while poor adherence is associated with depression (r = 0.21) (10). These factors may interact and reinforce each other in a vicious cycle.Given the multifaceted interaction among anxiety, social support, and adherence, integrated care models incorporating psychoeducation, peer support, and behavioral counseling may help break this cycle and improve mental health outcomes in diabetic patients.

Complications and disability significantly increased depression risk. A Danish cohort found T2DM complications raised depression/anxiety risk (HR = 1.77), with amputation having the strongest effect (HR = 2.16) (80). A meta-analysis confirmed increased risk in nephropathy patients (81). Mechanisms may involve chronic pain, loss of function, and treatment burden, reducing quality of life and increasing depression risk. Evidence regarding the impact of cardiovascular comorbidities like hypertension and coronary artery disease on depression among patients with diabetes remains inconclusive. While some studies suggest that comorbid conditions may exacerbate the psychological burden (82), others found no consistent association between depression and objective cardiovascular indicators (83, 84). A population-based study indicated that the history of cardiovascular events, rather than the mere presence of hypertension, was linked to depression (85). This suggests that the functional impact and severity of complications may be more critical than the simple presence of a comorbidity.

Treatment Regimen: Insulin use—especially combined oral and insulin therapy—was associated with higher depression risk. For example, A Korean study showed combined therapy patients had the highest depression rates (OR = 1.41), higher than insulin-only or oral-only users (86). Interestingly, this association was observed despite the lack of a significant relationship with HbA1c, suggesting the psychological impact may be related to the burdens of intensive treatment itself rather than glycemic control. Initiating insulin is often perceived by patients as a sign of disease progression or personal failure. Furthermore, the increased complexity, cost, and lifestyle rigidity associated with managing a combined regimen can be a source of distress (87, 88). This underlines the need for psychosocial support when initiating complex treatment regimens.

Fasting glucose ≥126 mg/dL was linked to higher depression risk; HbA1c was not. The significant association between elevated fasting blood glucose and depression is intriguing, though its interpretation is complex. This finding must be viewed in the context of an inconsistent literature regarding HbA1c; while some studies have reported positive correlations (89), others—including our pooled analysis of adjusted estimates—found no significant association after accounting for confounders (90). The discrepancy between FBG and HbA1c may reflect their distinct physiological correlates. FBG, particularly when measured in the morning after an overnight fast, may capture recent glycemic excursions and acute stress-related metabolic fluctuations involving cortisol and catecholamines (7, 89, 91). This mechanism is supported by evidence suggesting that acute glucose fluctuations (glycemic variability) are more strongly linked to negative psychological outcomes than mean glucose levels alone (91). In contrast, HbA1c represents average glycemic control over the preceding 2–3 months, and its long-term integrative nature may dilute the influence of acute psychological stress and is shaped by diverse clinical and behavioral factors, such as erythrocyte turnover, medication adherence, and diet. These findings underscore the importance of dynamic measures of glycemic variability in future research on the diabetes-depression nexus.

Although our pooled analysis indicated a non-significant trend toward lower depression risk in rural areas, this result should be interpreted with caution due to the small number of studies and wide confidence intervals. Prior meta-analyses suggest that urban–rural differences in depression are context-dependent. A global meta-analysis found that depression was significantly more prevalent in urban residents of developed countries, whereas no such association was observed in developing countries (92). Likewise, a systematic review and meta-analysis of older adults reported similar patterns (93). These findings imply that socioeconomic and community-level factors may underlie the heterogeneity of rural–urban differences.

This meta-analysis confirms the high comorbidity of depression in diabetes globally and identifies a suite of independent risk factors that contribute to this risk. The consistency of these findings across diverse settings underscores their potential utility in clinical practice. The implications of these results for developing targeted screening strategies are further elaborated in the conclusion.

Advantages and Limitations.

Advantages:

First, this study comprehensively included relevant literature on depression prevalence and associated risk factors among diabetic patients across multiple countries and regions.

Second, only multivariable logistic regression–adjusted ORs were included, which helped reduce the impact of confounding factors.

Third, the Hartung–Knapp adjustment was applied when heterogeneity exceeded 50%, yielding more conservative and reliable confidence intervals. This avoids the underestimation of uncertainty seen with traditional methods like DerSimonian–Laird in small or highly heterogeneous samples.

Fourth, the findings offer practical implications for clinical practice, including identifying high-risk individuals and informing stepped-care approaches.

Limitations:

As noted in the Discussion, the geographical distribution of included studies was uneven, with a predominance of research from Asia and Africa and fewer from Western countries and none from Latin America. This likely reflects global disparities in research funding and capacity, as well as differing regional priorities in public health research. It may limit the generalizability of our pooled prevalence estimate to high-income Western populations. However, this distribution also constitutes a unique strength of our study: it provides a much-needed synthesis of the evidence from low- and middle-income countries (LMICs), where the burden of diabetes is rising most rapidly and healthcare resources are often most strained. The risk factors identified (e.g., limited social support, unemployment) may be particularly relevant in these resource-limited settings. The absence of studies from Latin America highlights a significant gap in the literature that future research should aim to fill.

Second, only English-language publications were included, potentially omitting valuable data from non-English sources.

Third, substantial heterogeneity was present, partly due to differences in depression screening tools, which may have influenced the overall results.

Fourth, all studies were cross-sectional, limiting causal inferences.

## 5 Conclusion

This systematic review and meta-analysis found a high pooled prevalence of depression (35%) among patients with diabetes mellitus. More importantly, it identified a profile of specific, independent risk factors associated with significantly higher odds of depression, including sociodemographic (e.g., age ≤60 years, female gender, unemployment), psychosocial (e.g., anxiety, limited social support, poor medication adherence), and clinical factors (e.g., diabetic complications, insulin use, combination therapy, elevated fasting glucose).

Rather than reiterating the established need for routine screening, our findings provide an evidence-based framework for implementing risk-stratified screening protocols. Clinicians can use these identified risk factors to prioritize high-risk individuals (e.g., unemployed females with complications on insulin therapy) for more frequent and thorough assessment using standardized tools like the PHQ-9. This approach enables a move beyond blanket recommendations towards smarter, more efficient resource allocation, particularly in resource-constrained settings. For these high-risk groups, a stepped-care model—incorporating routine screening, brief interventions, and prompt referral—is essential.

Future large-scale longitudinal studies are needed to confirm the causal relationships suggested by our cross-sectional data and to refine the precision of these targeted prevention strategies.

## Data Availability

The original contributions presented in the study are included in the article/**Supplementary Material**. Further inquiries can be directed to the corresponding author.
