# Supplementary material for: Prevalence and risk factors of depression in patients with diabetes mellitus: a systematic review and meta-analysis
Source: Front Endocrinol (Lausanne). 2025 Oct 20;16:1660478. doi: 10.3389/fendo.2025.1660478 (PMC12580098; doi:10.3389/fendo.2025.1660478)
Supplement: Supplementary file 1 [file Table1.docx]

| **Section and Topic** | **Item #** | **Checklist item** | **Location where item is reported** |
| --- | --- | --- | --- |
| **TITLE** | | |  |
| Title | 1 | Identify the report as a systematic review. | Page1 |
| **ABSTRACT** | | |  |
| Abstract | 2 | See the PRISMA 2020 for Abstracts checklist. | Page1 |
| **INTRODUCTION** | | |  |
| Rationale | 3 | Describe the rationale for the review in the context of existing knowledge. | Page2 |
| Objectives | 4 | Provide an explicit statement of the objective(s) or question(s) the review addresses. | Page2 |
| **METHODS** | | |  |
| Eligibility criteria | 5 | Specify the inclusion and exclusion criteria for the review and how studies were grouped for the syntheses. | Page2 |
| Information sources | 6 | Specify all databases, registers, websites, organisations, reference lists and other sources searched or consulted to identify studies. Specify the date when each source was last searched or consulted. | Page2 |
| Search strategy | 7 | Present the full search strategies for all databases, registers and websites, including any filters and limits used. | Page2 |
| Selection process | 8 | Specify the methods used to decide whether a study met the inclusion criteria of the review, including how many reviewers screened each record and each report retrieved, whether they worked independently, and if applicable, details of automation tools used in the process. | Page2 |
| Data collection process | 9 | Specify the methods used to collect data from reports, including how many reviewers collected data from each report, whether they worked independently, any processes for obtaining or confirming data from study investigators, and if applicable, details of automation tools used in the process. | Page2 |
| Data items | 10a | List and define all outcomes for which data were sought. Specify whether all results that were compatible with each outcome domain in each study were sought (e.g. for all measures, time points, analyses), and if not, the methods used to decide which results to collect. | Page2 |
|  | 10b | List and define all other variables for which data were sought (e.g. participant and intervention characteristics, funding sources). Describe any assumptions made about any missing or unclear information. | Page2 |
| Study risk of bias assessment | 11 | Specify the methods used to assess risk of bias in the included studies, including details of the tool(s) used, how many reviewers assessed each study and whether they worked independently, and if applicable, details of automation tools used in the process. | Page2 |
| Effect measures | 12 | Specify for each outcome the effect measure(s) (e.g. risk ratio, mean difference) used in the synthesis or presentation of results. | Page2 |
| Synthesis methods | 13a | Describe the processes used to decide which studies were eligible for each synthesis (e.g. tabulating the study intervention characteristics and comparing against the planned groups for each synthesis (item #5)). | Page2 |
|  | 13b | Describe any methods required to prepare the data for presentation or synthesis, such as handling of missing summary statistics, or data conversions. | Page2 |
|  | 13c | Describe any methods used to tabulate or visually display results of individual studies and syntheses. | Page2 |
|  | 13d | Describe any methods used to synthesize results and provide a rationale for the choice(s). If meta-analysis was performed, describe the model(s), method(s) to identify the presence and extent of statistical heterogeneity, and software package(s) used. | Page2 |
|  | 13e | Describe any methods used to explore possible causes of heterogeneity among study results (e.g. subgroup analysis, meta-regression). | Page2 |
|  | 13f | Describe any sensitivity analyses conducted to assess robustness of the synthesized results. | Page2 |
| Reporting bias assessment | 14 | Describe any methods used to assess risk of bias due to missing results in a synthesis (arising from reporting biases). | Page2 |
| Certainty assessment | 15 | Describe any methods used to assess certainty (or confidence) in the body of evidence for an outcome. | N/A |
| **RESULTS** | | |  |
| Study selection | 16a | Describe the results of the search and selection process, from the number of records identified in the search to the number of studies included in the review, ideally using a flow diagram. | Page2-3 |
|  | 16b | Cite studies that might appear to meet the inclusion criteria, but which were excluded, and explain why they were excluded. | Page2-3 |
| Study characteristics | 17 | Cite each included study and present its characteristics. | Page4-5 |
| Risk of bias in studies | 18 | Present assessments of risk of bias for each included study. | Page4-5 |
| Results of individual studies | 19 | For all outcomes, present, for each study: (a) summary statistics for each group (where appropriate) and (b) an effect estimate and its precision (e.g. confidence/credible interval), ideally using structured tables or plots. | Page5 |
| Results of syntheses | 20a | For each synthesis, briefly summarise the characteristics and risk of bias among contributing studies. | Page3-5 |
|  | 20b | Present results of all statistical syntheses conducted. If meta-analysis was done, present for each the summary estimate and its precision (e.g. confidence/credible interval) and measures of statistical heterogeneity. If comparing groups, describe the direction of the effect. | Page3-5 |
|  | 20c | Present results of all investigations of possible causes of heterogeneity among study results. | Page6-7 |
|  | 20d | Present results of all sensitivity analyses conducted to assess the robustness of the synthesized results. | Page7 |
| Reporting biases | 21 | Present assessments of risk of bias due to missing results (arising from reporting biases) for each synthesis assessed. | N/A |
| Certainty of evidence | 22 | Present assessments of certainty (or confidence) in the body of evidence for each outcome assessed. | N/A |
| **DISCUSSION** | | |  |
| Discussion | 23a | Provide a general interpretation of the results in the context of other evidence. | Page9-11 |
|  | 23b | Discuss any limitations of the evidence included in the review. | Page11 |
|  | 23c | Discuss any limitations of the review processes used. | Page11 |
|  | 23d | Discuss implications of the results for practice, policy, and future research. | Page11 |
| **OTHER INFORMATION** | | |  |
| Registration and protocol | 24a | Provide registration information for the review, including register name and registration number, or state that the review was not registered. | Page2 |
|  | 24b | Indicate where the review protocol can be accessed, or state that a protocol was not prepared. | Not prepared |
|  | 24c | Describe and explain any amendments to information provided at registration or in the protocol. | N/A |
| Support | 25 | Describe sources of financial or non-financial support for the review, and the role of the funders or sponsors in the review. | Page12 |
| Competing interests | 26 | Declare any competing interests of review authors. | Page11 |
| Availability of data, code and other materials | 27 | Report which of the following are publicly available and where they can be found: template data collection forms; data extracted from included studies; data used for all analyses; analytic code; any other materials used in the review. | Supplementary  file |

*From:*  Page MJ, McKenzie JE, Bossuyt PM, Boutron I, Hoffmann TC, Mulrow CD, et al. The PRISMA 2020 statement: an updated guideline for reporting systematic reviews. BMJ 2021;372:n71. doi: 10.1136/bmj.n71. This work is licensed under CC BY 4.0. To view a copy of this license, visit <https://creativecommons.org/licenses/by/4.0/>

Appendix 2 Literature search strategy

| Database | Combined Search Code |
| --- | --- |
| PubMed | (((("Diabetes Mellitus"[MeSH Terms]) OR ("Diabetes Mellitus, Type 1"[MeSH Terms])) OR ("Diabetes Mellitus, Type 2"[MeSH Terms]) AND ((humans[Filter]) AND (english[Filter]))) AND ((((("Depression"[MeSH Terms]) OR ("Depressive Symptoms"[Title/Abstract])) OR ("Emotional Depression"[Title/Abstract])) OR ("Major Depressive Disorder"[Title/Abstract])) OR ("Mood Disorders"[Title/Abstract]) AND ((humans[Filter]) AND (english[Filter]))) AND ((humans[Filter]) AND (english[Filter]))) AND ((((((((((((((((("prevalence"[MeSH Terms]) ) OR ("incidence*"[MeSH Terms])) OR ("epidemiology"[MeSH Terms])) OR ("Cross-Sectional Studies"[MeSH Terms])) OR ("Cohort Studies"[MeSH Terms])) OR ("Risk Factors"[MeSH Terms])) OR ("Analysis, Cross-Sectional"[Title/Abstract])) OR ("Cross Sectional Analysis"[Title/Abstract])) OR ("Cross-Sectional Survey"[Title/Abstract])) OR ("Disease Frequency Surveys"[Title/Abstract])) OR ("Prevalence Studies"[Title/Abstract])) OR ("Analysis, Cohort"[Title/Abstract])) OR ("Health Correlates"[Title/Abstract])) OR ("Population* at Risk"[Title/Abstract])) OR ("Risk Scores"[Title/Abstract])) OR ("associated factors"[Title/Abstract]) AND ((humans[Filter]) AND (english[Filter]))) |
| Web of Science Core Collection | #1"Diabetes Mellitus" OR "Diabetes Mellitus, Type 1" OR "Diabetes Mellitus, Type 2" (Topic)  #2"Depression" OR "Depressive Symptoms" OR "Emotional Depression" OR "Major Depressive Disorder" OR "Mood Disorders" (Topic)  #3"prevalence" OR "incidence*" OR "epidemiology" OR "Cross-Sectional Studies" OR "Cohort Studies" OR "Risk Factors" OR "Analysis, Cross-Sectional" OR "Cross Sectional Analysis" OR "Cross-Sectional Survey" OR "Disease Frequency Surveys" OR "Prevalence Studies" OR "Analysis, Cohort" OR "Health Correlates" OR "Population* at Risk" OR "Risk Scores" OR "associated factors" (Topic)  #4 #3 AND #2 AND #1 and English (Languages) |
| Embase | #1 'diabetes mellitus'/exp OR 'diabetes mellitus, type 1'/exp OR 'diabetes mellitus, type 2'/exp  #2 'depression'/exp OR 'depressive symptoms':ti,ab,kw OR 'emotional depression':ti,ab,kw OR 'major depressive disorder':ti,ab,kw OR 'mood disorders':ti,ab,kw  #3 'prevalence'/exp OR 'incidence*' OR 'epidemiology'/exp OR 'cross-sectional studies'/exp OR 'cohort studies'/exp OR 'risk factors'/exp OR 'analysis, cross-sectional':ti,ab,kw OR 'cross sectional analysis':ti,ab,kw OR 'cross-sectional survey':ti,ab,kw OR 'disease frequency surveys':ti,ab,kw OR 'prevalence studies':ti,ab,kw OR 'analysis, cohort':ti,ab,kw OR 'health correlates':ti,ab,kw OR 'population* at risk':ti,ab,kw OR 'risk scores':ti,ab,kw OR 'associated factors':ti,ab,kw  #4 #1 AND #2 AND #3  #5 #1 AND #2 AND #3 AND [article]/lim AND ([adult]/lim OR [young adult]/lim OR [middle aged]/lim OR [aged]/lim OR [very elderly]/lim) AND [humans]/lim AND [english]/lim AND [clinical study]/lim |
| Cochrane Library | #1 MeSH descriptor: [Diabetes Mellitus] explode all trees  #2 (diabete*):ti,ab,kw  #3 #1 OR #2  #4 MeSH descriptor: [Depression] explode all trees  #5 ((Emotional Depression):ti,ab,kw OR (Depressive Symptoms):ti,ab,kw OR (Depressive Symptom):ti,ab,kw OR (Symptom, Depressive):ti,ab,kw OR (Depression, Emotional)):ti,ab,kw  #6 #4 OR #5 41808  #7 MeSH descriptor: [Prevalence] explode all trees  #8 ((Period Prevalence):ti,ab,kw OR (Prevalence, Period):ti,ab,kw OR (Period Prevalences):ti,ab,kw OR (Prevalences):ti,ab,kw OR (Point Prevalence):ti,ab,kw OR (Prevalence, Point):ti,ab,kw OR (Point Prevalences)):ti,ab,kw  #9 #7 OR #8  #10 MeSH descriptor: [Risk Factors] explode all trees  #11 ((Risk Factor):ti,ab,kw OR (Factor, Risk):ti,ab,kw OR (Risk Factor Scores):ti,ab,kw OR (Score, Risk Factor):ti,ab,kw OR (Score, Risk):ti,ab,kw OR (Risk Scores):ti,ab,kw OR (Risk Factor Score):ti,ab,kw OR (Risk Score):ti,ab,kw OR (Risk Factor, Social):ti,ab,kw OR (Factors, Social Ris):ti,ab,kw OR (Social Risk Factor):ti,ab,kw OR (Risk Factors, Social):ti,ab,kw OR (Factor, Social Risk):ti,ab,kw OR (Social Risk Factors):ti,ab,kw OR (Health Correlates):ti,ab,kw OR (Correlates, Health):ti,ab,kw OR (Populations at Risk):ti,ab,kw OR (Population at Risk)):ti,ab,kw  #12 #10 OR #11  #13 #9 OR #12  #14 #3 AND #6  #15 #13 AND #14 |
| ProQuest | #1 (title("diabetes mellitus" OR "Diabetes Mellitus, Type 1" OR "Diabetes Mellitus, Type 2" OR "Diabete*") OR abstract("diabetes mellitus" OR "Diabetes Mellitus, Type 1" OR "Diabetes Mellitus, Type 2" OR "Diabete*")) AND la.exact("English")  Databases: Health & Medical Collection  #2 (title(Depression) OR abstract("Depressive Symptoms" OR "Emotional Depression" OR "Major Depressive Disorder" OR "Mood Disorders")) AND la.exact("English")  Databases: Health & Medical Collection  #3 (title("prevalence" OR "incidence*" OR "epidemiology" OR "Risk Factors") OR abstract("Analysis, Cross-Sectional" OR "Cross Sectional Analysis" OR "Cross-Sectional Survey" OR "Disease Frequency Surveys" OR "Prevalence Studies" OR "Analysis, Cohort" OR "Health Correlates" OR "Population* at Risk" OR "Risk Scores" OR "associated factors")) AND la.exact("English")  Databases: Health & Medical Collection  #4 #1 AND #2 AND #3  Databases: Health & Medical Collection |

Appendix 3 Full details of the 39 included studies are provided

| Author+year | Country | Study Design | Setting | Survey period | N | Age | Assessment | Types | Duration | n | Prevalence  (%) | JBI1 | JBI2 |
| --- | --- | --- | --- | --- | --- | --- | --- | --- | --- | --- | --- | --- | --- |
| Mohamed Abd-Elgawad2023 | Egypt | cross-sectional | hospital | 2020.11-2020.12 | 679 | 51.67±12.59 | HADS≥8 | Type1,Type2 | / | 232 | 34.17 | L | L |
| Shahad Abduljalil Abualhamael2024 | KSA | cross-sectional | Faculty of Medicine | 2021.09-2022-03 | 251 | 50.1±14.5 | DASS-21≥10 | Type2 | / | 124 | 49.40 | H | L |
| Hesham Abuhegazy2022 | KSA | cross-sectional | hospital | 2017.03.01-2017.06.30 | 350 | 61.4±13 | PHQ-9＞10 | Type2 | / | 128 | 36.57 | M | L |
| Seid Yimam Ali | Ethiopia | cross-sectional | hospital | 2022.10.03-2022.11.13 | 263 | 50.21±14.81 | PHQ-9≥5 | Type2 | / | 124 | 47.15 | M | L |
| Abdullahi S. Aminu2017 | India | cross-sectional | community | 2016.01-2016.06 | 200 | 63±11.478 | PHQ-9≥5 | Type2 | 8.00 | 75 | 37.50 | M | L |
| Muhammad Atif2018 | Pakistan | cross-sectional | hospital | 2015.12.02-2016.02.28 | 400 | 64±5.5 | GDS-15≥5 | Type2 | / | 270 | 67.50 | M | L |
| Gedion Asnake Azeze2020 | Ethiopia | cross-sectional | hospital | 2019.11.01-2019.12.15 | 410 | 47.4±9.6 | PHQ-9≥5 | Type2 | / | 120 | 29.27 | L | L |
| anteneh Messele Birhanu2016 | Ethiopia | cross-sectional | hospital | 2014.03.10-2014.05.30 | 415 | 44.2±15.8 | PHQ-9≥5 | Type1,Type2 | / | 64 | 15.42 | L | L |
| Habtamu Birhanu2022 | Ethiopia | cross-sectional | hospital | 2021.04-2021.05 | 310 | 47.22 | PHQ-9≥10 | Type1,Type2 | / | 129 | 41.61 | L | L |
| Tania Dehesh2020 | Iran | cross-sectional | hospital | 2018.08-2018.11 | 1500 | 47.12±12.52 | BDI-Ⅱ≥18 | Type2 | / | 885 | 59.00 | L | L |
| Mohamed Ebrahim2021 | Ethiopia | cross-sectional | hospital | 2020.06.01-2020.07.01 | 401 | 48.6±13.2 | PHQ-9≥5 | Type1,Type2 | / | 196 | 48.88 | L | L |
| Mohamed Hassan Elnaem2025 | Indonesia/Malaysia | cross-sectional | Online | 2022.10-2023.04 | 606 | / | PHQ-9≥10 | Type2 | / | 343 | 56.60 | M | L |
| Nigus Alemnew Engidaw2020 | Ethiopia | cross-sectional | hospital | 2019.02.08-2019.04.08 | 403 | 46.4±13.3 | PHQ-9≥5 | Type1,Type2 | / | 86 | 21.34 | L | L |
| Annie C. H. Fung2018 | China | cross-sectional | hospital | 2013.02-2013.12 | 325 | 69±5.93 | GDS-15≥7 | Type2 | 12±8.3 | 42 | 12.92 | H | L |
| Malgorzata Gorska-Ciebiada2014 | Poland | cross-sectional | hospital | / | 276 | 73.6±4.8 | GDS-30≥10 | Type2 | 8.69±6.23 | 82 | 29.71 | H | L |
| Sheikh Mohammed Shariful Islam2015 | Bangladesh | cross-sectional | hospital | 2013.09-2014.07 | 515 | 49.94±10.21 | PHQ-9≥5 | Type2 | / | 319 | 61.94 | H | L |
| Firdous Jahan2011 | Pakistan | cross-sectional | hospital | / | 320 | 54.54±12.50 | self-reported validated questionnaire≥9 | Type1,Type2 | 10.49±7.34 | 56 | 17.50 | M | L |
| Mihyun Jeong2021 | Korean | cross-sectional | KNHANES | 2014+2016+2018 | 1529 | 63.34 ± 0.68 | PHQ-9≥10 | Type1,Type2 | / | 149 | 9.74% | L | L |
| Ashmita Karki2024 | Nepal | cross-sectional | community | / | 481 | 54±9.41 | PHQ-9≥5 | Type2 | / | 123 | 25.57 | L | L |
| Kankana Karpha2022 | India | cross-sectional | hospital | 2022.04-2022.07 | 152 | 55.13±7.70 | PHQ-9≥5 | Type1,Type2 | 7.50±3.57 | 60 | 39.47 | L | L |
| Nuket Bayram Kayar2017 | Turkey | cross-sectional | hospital | / | 154 | 54.8±9.1 | SCID-I scale | Type2 | / | 28 | 18.18 | M | L |
| Steven M. Kogan2007 | America | cross-sectional | community | / | 200 | 52.46±6.687 | CES-D≥16 | Type2 | 5.85±2.91 | 72 | 36.00 | L | L |
| Rehanguli Maimaitituerxun2023 | China | cross-sectional | hospital | 2021.03-2021.12 | 496 | 59.57±9.92 | HADS-D≥8 | Type2 | 11.21±7.75 | 135 | 27.22 | L | L |
| Makda Abate Belew2023 | Ethiopia | cross-sectional | hospital | 2022.01.07-2022.02.10 | 426 | / | PHQ-9≥5 | Type1,Type2 | / | 203 | 47.65 | L | L |
| Eva O. Melin2017 | Sweden | cross-sectional | hospital | 2009.03-2009.12 | 148 | 46.33±5.30 | HADS-D≥8 | Type1 | 23.67±7.76 | 17 | 11.49 | H | L |
| Nelda Mier2008① | Mexico | cross-sectional | hospital | 2004-2005 | 200 | 55.8±12.2 | CES-D≥16 | Type2 | 10.1±9.9 | 81 | 40.50 | L | L |
| Nelda Mier2008② | America | cross-sectional | hospital | 2004-2005 | 172 | 57.8±14.1 | CES-D≥16 | Type2 | 11.0±10.1 | 67 | 38.95 | L | L |
| Nur Adam Mohamed2024 | Somalia | cross-sectional | hospital | 2023.11-2024.01 | 360 | 46.7±12.5 | DASS-21≥10 | Type2 | / | 161 | 44.72 | L | L |
| Lili Husniati Yaacob2012 | Malaysia | cross-sectional | hospital | 2007.11.01-2008.03.01 | 260 | 50.9±6.3 | HADS-D≥9 | Type2 | 7.3±5.5 | 54 | 20.77 | L | L |
| Mussa R. Mussa2023 | Tanzania | cross-sectional | hospital | 2019.03-2019.07 | 267 | 50±14 | PHQ-9≥5 | Type1,Type2 | / | 194 | 72.66 | L | L |
| Kabtamu Nigussie2023 | Ethiopia | cross-sectional | hospital | 2022.03-2022.04 | 416 | 53.5±12.59 | HADS≥8 | Type2 | / | 176 | 42.31 | L | L |
| Hina Sharif2023 | Pakistan | cross-sectional | community | 2022.08-2022.09 | 493 | 48.3±12.8 | PHQ-9≥5 | Type1,Type2 | / | 152 | 30.83 | L | L |
| Avinash K. Sunny2019 | Nepal | cross-sectional | community | 2015.09-2016.08 | 278 | 54.3±11.2 | BDI-Ⅱ≥16 | Type2 | / | 63 | 22.66 | L | L |
| Waleed M Sweileh2014 | Palestine | cross-sectional | hospital | 2012.04-2012.08 | 294 | 59±10.56 | BDI-Ⅱ≥16 | Type2 | 12±8.15 | 120 | 40.82 | L | L |
| Thitiphan Thaneerat2009 | Thailand | cross-sectional | hospital | 2008.06-2008.12 | 250 | 62.58±10.41 | HADS-D≥8 | Type2 | 12.84±8.35 | 70 | 28.00 | M | L |
| Nhu Minh Hang Tran2021 | Vietnam | cross-sectional | hospital | 2018.09-2019.04 | 216 | 64.73±13.68 | PHQ-9≥10 | Type2 | / | 50 | 23.15 | M | L |
| Allan Oliver Dampil2019 | Philippines | cross-sectional | hospital | 2018.05-2018.12 | 476 | 58.3±11.8 | PHQ-9≥5 | Type2 | / | 386 | 81.09 | L | L |
| Yiting Wang2016 | America | cross-sectional | NHANES | 2005-2012 | 2182 | / | PHQ-9≥10 | Type2 | / | 256 | 11.73 | L | L |
| Weijun Zhang2015 | China | cross-sectional | hospital | 2012.03.01-2012.05.31 | 412 | 59.77±12.48 | BDI-Ⅱ≥14 | Type2 | 8.93±6.51 | 142 | 34.47 | L | L |

Notes. N:Sample Size;Age:mean±SD (y);Assessment:Depression Assessment and Cut-off Score;Types:Types of Diabetes;Duration:Duration of DM mean (SD) / range;n:Presence of Depression;JBI:Joanna Briggs Institute;L:LOW;M:Moderate;H:HIGH

Appendix 4. Bubble plot of multivariable meta-regression examining the effects of mean age, survey year, and geographic region on depression prevalence among diabetic patients. Each circle represents an individual study. The size of the circle is proportional to the survey year (larger circles indicate more recent years). The color of the circle represents the geographic region (continent). The solid black line represents the predicted regression line for the effect of mean age, holding other variables constant; the shaded area represents its 95% confidence interval.





Appendix 5. Results of leave-one-out sensitivity analysis showing the robustness of the pooled prevalence estimate of depression in diabetic patients
